# Marketing by live streaming: How to interact with consumers to increase their purchase intentions

**DOI:** 10.3389/fpsyg.2022.933633

**Published:** 2022-08-25

**Authors:** Feng Liu, Yan Wang, Xiaoxu Dong, Huawei Zhao

**Affiliations:** ^1^Business School, Shandong University, Weihai, China; ^2^School of Business, Shandong University of Political Science and Law, Jinan, China; ^3^College of Business and Economics, Shanghai Business School, Shanghai, China

**Keywords:** live streaming, social presence, consumer conformity, purchase intention, search product, experience product

## Abstract

Live streaming e-commerce, as a kind of new information technology-based business model, is currently the most popular marketing approach, especially in China. This research divides live streaming interactions into three dimensions, interactions for obtaining product information (IPI), interactions for grasping the purchase dynamics of others (IPD), and interactions for obtaining monetary incentives (IMI), and proposes a comprehensive framework to examine whether live streaming interactions with consumers promote both social presence and consumer conformity, and thereby enhance their purchase intentions. Covariance-based structural equation modeling (CB-SEM) with AMOS was conducted to analyze data collected from 576 Chinese consumers. The main findings are that, first, both IPI and IPD can exert a direct impact on social presence and consumer conformity; second, IMI has a positive impact only on social presence; third, among the three dimensions of interactions, both IPI and IPD tend to increase purchase intention through social presence and consumer conformity, while IMI increases purchase intention only *via* social presence. Furthermore, given the differences between experience and search products, the results of the multigroup analysis indicate inconsistent path coefficients between the two product groups. This study provides a novel perspective on live streaming e-commerce with evidence on how interactions matter in driving purchase intentions, enriches the content of live streaming e-commerce literature and explores the practical implications for marketing managers are looking for marketing by live streaming.

## Introduction

The commercial application of next-generation information technology has brought wide-ranging business changes, and the touch points of customer experience have expanded from limited offline stores to the digital space (Hoyer et al., 2020). Live streaming, as novel information technology, has been an embedded live function of e-commerce platforms that are operated by mainstream e-commerce enterprises, such as Taobao.com and Jingdong.com (Wongkitrungrueng and Assarut, 2018; Cunningham et al., 2019; Sun et al., 2019; Park and Lin, 2020; Xu and Ye, 2020; Guo et al., 2021). Compared with traditional e-commerce, live-streaming e-commerce has become appealing to marketers because of its powerful interactions (Hu and Chaudhry, 2020; Kang et al., 2020; Xu et al., 2020). As of December 2020, the number of live-streaming e-commerce consumers in China had climbed to 388 million, and the sales promotion effect of live-streaming e-commerce as a novel strategy has been featured by numerous e-commerce firms, such as Alibaba, Byte Dance, Kuaishou, and Pinduoduo. For example, Taobao Live (a live stream e-commerce platform of Alibaba) was reported to generate nearly 50 billion US dollars in gross merchandise volume (GMV) in 2020 (Pandaily, 2021).

In contrast to the one-way efforts (i.e., streamer to consumer) made by other information technologies to improve consumer experience (such as intelligent chat assistants, virtual/augmented reality, and intelligent service robots), live streaming realizes bidirectional communication between streamers and consumers in a real-time interactive way, thus providing consumers with an immersive shopping experience and an emotional value in interpersonal interactions (Haimson and Tang, 2017; Wohn et al., 2018). In live-streaming rooms, streamers can display product information in diversified forms, including but not limited to offering product close-ups, model displays, and promotions, which reduce the uncertainty of consumers (Kozlenkova et al., 2017). In particular, streamers' opinions eliminate consumers' concerns and instill confidence in them. At the same time, consumers improve their social presence through real-time interaction with streamers and other viewers, thus gaining pleasant emotional experiences (Chen and Lin, 2018). Nowadays, because of the characteristics of real-time interaction, the technology of live streaming has been widely applied to online marketing by enterprises to create higher conversion rate and better sales (Hu and Chaudhry, 2020). However, although many studies have investigated the mechanism of influence of live-streaming e-commerce on consumers' purchase intention (Wongkitrungrueng and Assarut, 2018; Sun et al., 2019; Park and Lin, 2020; Xue et al., 2020; Ma, 2021a,b), only a few of them involve the unique feature of live streaming e-commerce (i.e., interactions), making it unclear how live streaming interactions promote consumers' purchase intentions.

This research seeks to conduct an in-depth analysis of interaction-related behaviors in live-streaming e-commerce. Prior literature argued that there are two levels of interactivity: person interactivity and machine interactivity (Hoffman and Novak, 1996). Live streaming can provide the above two levels of interactivity: person interactivity through communication between the streamer and consumers in the live streaming room, and the use of embedded hyperlinks allows consumers to purchase products in the live-streaming room to generate machine interactivity. On this basis, many scholars classified live streaming interaction (LSI) as streamer-consumer interactions, consumer-consumer interactions, or machine-consumer interaction (Chen and Lin, 2018; Jiang et al., 2019). However, with integration of live-streaming e-commerce, richer shopping experience has been created (Sun et al., 2019), and the elements and forms of live streaming interactions can no longer be simply divided by participants. Therefore, this study attempts to reclassify LSI and find a new research perspective on the interactions.

Different from previous studies, this study focuses on reclassifying LSI from the perspective of consumer participation motivation. First of all, the computer-mediated communication (CMC) interaction model provides the theoretical premise for our interaction framework. CMC is generally considered to be an emerging model of information search, transmission, processing and interpersonal communication through the Internet (Walther, 1996; Derks et al., 2008; Yao and Ling, 2020). Relevant studies have confirmed the effectiveness of CMC in emotional transmission (Sasaki and Ohbuchi, 1999; Walther et al., 2005; Hancock et al., 2008; Grondin et al., 2019). In live-streaming e-commerce, some nonverbal cues related to emotion are shown to consumers, such as streamers' facial expressions, gestures, and tones, so as to generate emotional contagion among participants, which is the precondition of LSI. Second, information gathering, social interaction, and online shopping are regarded as the main motivations for consumers to watch live streaming (Wongkitrungrueng and Assarut, 2018; Sun et al., 2019; Xu and Ye, 2020). Consumers can obtain instant electronic word of mouth by participating in the evaluation of products by streamers and other consumers while also conducting observational learning from the actual purchase behavior of others (Chen et al., 2011; Men and Zheng, 2019). Moreover, consumers make trade-offs between hedonic consumption and product prices, and special offers during live streaming can bring hedonic benefits to consumers (Alba and Williams, 2013; Xu et al., 2020). Therefore, based on consumers' motivation to participate in live streaming, our interaction framework is developed with three subdimensions, which are interactions for obtaining product information (IPI), interactions for grasping the purchase dynamics of others (IPD), and interactions for obtaining monetary incentives (IMI). Overall, we suggest the above categorization of three interactions, driven by practical experience, can more clearly and vividly describe the activities of interactions of live-streaming e-commerce.

By live streaming, consumers can participate in an immersive experience by watching streamers' product presentations, interacting with streamers, reading consumer discussions, and enjoying platform discounts. This immersive experience increases consumers' social presence (Mou et al., 2017). The concept of social presence has been widely adopted in online interaction and consumer research, and discusses the influence of social presence on perceived value (Fang et al., 2018), consumers' trust (Gefen and Straub, 2004; Jiang et al., 2019), and purchase intention (Botha and Reyneke, 2016; Lu et al., 2016; Liu et al., 2019). However, only a few studies involve the high real-time and highly interactive situation of live-streaming e-commerce. Meanwhile, the swarming of other consumers prompts consumers to follow and automatically internalize themselves as group members, which promotes conformity, whereas previous research on consumer conformity mainly focus on offline consumption scenes and online graphic consumption scenes (Laporte et al., 2010; Park and Feinberg, 2010), and the conformity psychology and behavior in the context of live streaming still need to be explored. To sum up, previous theories and studies cannot completely explain and predict consumer behavior under the live-streaming e-commerce mode. It is necessary to explore the marketing benefits of LSI and determine how consumers may form their purchase intentions through social presence and consumer conformity.

Despite the importance of considering the roles of different product types, this factor has been largely ignored in the existing research when studying live streaming e-commerce. Hence, it is crucial to integrate product type (i.e., search and experience products) into our study to deeply explore LSI. In line with Nelson (1970), products fall into two categories: search and experience products. Consumers show varying degrees of difficulty in assessing the attributes of experience products and search products; specifically, the attributes of search products, i.e., cell phones, televisions, laptops, and books, are objective and easy to access, while the key attributes of experience products (e.g., clothing, shoes, food, and cosmetics) need to be evaluated through more external cues (Bei et al., 2004; Luan et al., 2016). In the context of live-streaming e-commerce, LSI provides consumers with more specific product information and purchase reference, thus reducing barriers to buying products online, especially for experience products that cannot be accurately assessed before purchase (Moon et al., 2008). Therefore, it seems important to examine how product types affect consumers' purchase decision processes to understand the effectiveness of LSI. In this regard, this study also extends the understanding of how consumers' underlying motivations for purchasing decisions differ in the context of live-streaming e-commerce depending on product searching and experiencing.

By adopting the stimulus-organism-response (S-O-R) theory as a guiding theoretical lens, this study enriches the literature on live-streaming e-commerce by developing a research model to examine whether LSI promotes both social presence and consumer conformity and enhances consumers' purchase intentions. Specifically, first, this research conducts an in-depth analysis of interaction-related behaviors in live-streaming e-commerce, including IPI, IPD, and IMI. Second, this research reveals the formation causes of social presence and consumer conformity in live-streaming e-commerce from the perspective of the above interactions and empirically investigates the relationship between social presence and consumer conformity. Third, this study introduces product type as a moderating variable by examining the impact of search and experience products on live-streaming e-commerce and provides a new marketing perspective for industry practitioners.

Compared with previous literature, this article made four main contributions. First, compared to past studies on interactions in this context, which were mostly classified according to the object of interactions, this study developed a new measurement for LSI and proposed three dimensions: IPI, IPD, and IMI. In particular, we employed IMI as a dimension of monetary incentive interactions and verified its role in increasing social presence. Second, this study extended the understanding of consumers' purchase intention in the context of live streaming. Third, this study applied and integrated the two theories of social presence and consumer conformity, and explained the potential incentives for consumers' purchases in live-streaming e-commerce. Fourth, this study paid more attention to product factors, which had been neglected in previous studies. Overall, this study contributes to the live streaming e-commerce literature by advancing the understanding of LSI and exploring the practical implications for marketing managers who are looking for marketing by live streaming.

## Literature review and hypotheses

### Stimulus-organism-response theory

The S-O-R theory, proposed by Mehrabian and Russell (1974), was initially used to explain the impact of the environment on human psychology and behavior. Three crucial elements are included in this theory. Stimulus (S) stands for antecedent variables, including various situational factors; organism (O) refers to an individual's emotional or cognitive response to the S; response (R) is final motivations and behaviors based on affective and cognitive states. The use of the S-O-R theory as a theoretical framework for this research is advantageous for two reasons. First, this model provides the theoretical basis for the understanding of consumer behavior and has been widely applied to examine consumers' behaviors in e-commerce, such as purchase intention (Guo et al., 2021), online repurchase intention (Zhu et al., 2020), and impulse buying behaviors (Chen and Yao, 2018). Second, the S-O-R theory allows us to capture the unique dimensions of real-time social interaction in live-streaming e-commerce and construct a dynamic mechanism to present how LSI influences customer purchase intentions. Overall, the S-O-R theory fits our research questions and contributes to hypothesis development. Specifically, the interactions in the context of live-streaming e-commerce as a strong stimulus influence individuals' emotional or cognitive response by way of social presence and consumer consistency, and further influence purchase intentions.

### Interaction factors for purchase intention

The interaction factor is regarded as a key driver for purchase intention. This study mainly reviews the literature on linkage between various interactions and purchase intention. Our research builds primarily on two streams, one that examines how social interactions affect consumer purchase behaviors. For example, a case of group buying indicated that a seller could benefit from social interaction because of interpersonal information/knowledge sharing (Jing and Xie, 2011). Similarly, Kim et al. (2020), based on a survey on Korean consumers, concluded that increased social interactions on an online platform could promote sales performance. Furthermore, based on an investigation of YouTube and Instagram users, Sokolova and Kefi (2020) argued that para-social interaction (i.e., the relationship between a spectator and a performer) is positively related to customers' purchase intention.

Another stream determines how to interact with customers to influence purchase intention. The research on this stream can be classified into two broad categories. The first is related to the interpersonal interaction between sellers and buyers, with some dimensions of interaction behavior (e.g., perceived expertise, perceived similarity, perceived likeability, response volume, topic relevance, and response richness) found to affect consumer purchase intention (Chen et al., 2021; Kim et al., 2021). The second category involves the interaction among consumers; i.e., Chang and Dong (2016) found that consumer interaction (interaction frequency, interestingness, decentration, and reciprocal swap) can enhance purchase intention by formation of cognitive trust and emotional trust.

The influence of LSI on purchase intention is studied for three main reasons. First, live-streaming e-commerce as a novel business model has received a great deal of attention from scholars and practitioners. Second, because the success of live-streaming e-commerce depends on live interactions between streamers and customers and customer engagement, it is crucial to explore the effects of LSI on consumers' purchase intentions. Third, the multiple facets of LSI need to be extended and enriched, because little is known about how the LSI between streamers and consumers or among consumers impacts purchase intention. Based on the foregoing, this study aims to explore how LSI enhances customers' purchase intentions.

### Live-streaming interactions

Establishing a positive and effective interaction between sellers and consumers will help increase consumers' pleasure and experience and will assist in achieving enterprises' marketing goals (Kostopoulos et al., 2020; Zhang et al., 2020). Prior studies have tried to classify interactions from different perspectives (Steuer, 1992; Burton and Soboleva, 2011; Li, 2019; Xu and Ye, 2020). Among them, the most representative classification is to divide interactions according to interactive content. For example, in a study on online agents, interactions are divided into social interactions, human-computer interactions, and information interactions (Kohler et al., 2011). Interactions have undergone further development in live-streaming e-commerce, and both the form and connotations of interactions have been greatly enriched; therefore, more research on LSI is needed. Existing studies have already noted that because of changes in technology, LSI involves more participants than other forms of e-commerce, and that the objects involved in the interactions have also changed (Chen and Lin, 2018; Jiang et al., 2019; Xue et al., 2020). LSI was reclassified as anchor-consumer interaction, consumer-consumer interaction, machine-consumer interaction, or merchant-consumer interactions (Chen and Lin, 2018; Jiang et al., 2019). Rather than employ classifications according to the participants in interactions, this study attempts to reclassify LSI and find a new research perspective on interactions. As mentioned in Introduction, the current study divides LSI into the following three aspects: IPI (i.e., interactions for obtaining product information), IPD (i.e., interactions for grasping the purchase dynamics of others), and IMI (i.e., interactions for obtaining monetary incentives). Overall, we suggest that the above categorization of three interactions can more clearly and vividly describe the effect of interactions on live-streaming e-commerce.

### Social presence

Social presence was first conceptualized as “the degree of other people's salience in interactions and the consequent salience of the interpersonal relationship” (Short et al., 1976, p.65). In electronic commerce and online consumer behavior research, social presence refers to the feeling of being with others in virtual interactions. Thus, users feel a sense of warmth and enjoy social communication as if they were in an actual environment (Hassanein and Head, 2007). In the context of e-commerce, social presence can affect consumers' online shopping experience, which is a key behavior premise in virtual shopping settings (Burton and Soboleva, 2011; Shin and Shin, 2011; Kim, 2015; Ma, 2021b). Moreover, the social presence conveyed by a website influences behavioral intention by enhancing the perception of pleasure and usefulness (Shen, 2012). Furthermore, Pelet et al. (2017) pointed out that a social platform is a mature environment that forms social presence. This is because some features of social platforms, such as perceived interaction, enable consumers to experience virtual presence in an online environment (Mollen and Wilson, 2010). Interactions on social platforms can deliver various information and enable consumers to feel close ties between themselves, thus representing social presence (Jiang et al., 2019). Based on the foregoing, interactions on a social platform are considered to be the most important factor.

IPI enables consumers to obtain more product information through live product trials and detailed product explanations, which can increase social presence. The difference between live-streaming e-commerce and traditional e-commerce is that live streaming not only provides product information through text and pictures but also through interactions with streamers (Xue et al., 2020). During live streaming, buyers may use information cues (e.g., product information) to support decision-making processes (Dodds et al., 1991). On such a platform, consumers can actively participate in interactions to obtain detailed product information. Therefore, consumers can perceive that the member with whom they are communicating is a real person, which leads them to perceive interactive social presence (Li, 2019; Sun et al., 2019). Through facial expressions and body language, streamers can give consumers a full understanding and make it easier for both sides to form a sense of identity and belonging in the virtual space. Furthermore, online interactions provide consumers with a sense of immersion into a virtual, unreal world (Hamilton et al., 2016; Hudson et al., 2019). Overall, such an online interaction is akin to face-to-face communication between streamers and consumers. In this virtually created space, both sides psychologically apprehend the other's existence, which meets the social needs of both parties and fosters effective communication that leads to the formation of a sense of presence. Hence, the following hypothesis is proposed:

**H1a: Interactions for obtaining product information (IPI) have a positive impact on social presence**.

IPD equates to frequently interacting with other consumers in the same live streaming room. Consumers, when communicating and sharing information with other consumers, are not isolated as individuals but are instead situated in a type of social existence that increases their familiarity and enables them to discern a sense of cordiality in continuous, in-depth interactions. We propose that IPD affects social presence in three ways. First, based on the statement of Kim et al. (2013), p. 171; that behavioral commitment is “the degree of perceived interdependency, connection, and responsiveness of the other person to the observer's actions”, IPD can enhance social presence through other viewers' purchase behaviors. Second, the communication between viewers and streamers, as constructive interaction, can facilitate trust, thereby increasing social presence (Gefen and Straub, 2004). Third, real-time discussions with other participants in IPD can enhance the perceived social presence by responding to product content and sellers (Hassanein and Head, 2005; Fang et al., 2018; Sun et al., 2019). Hence, the following related hypothesis is proposed:

**H1b: Interactions for grasping the purchase dynamics of others (IPD) have a positive impact on social presence**.

IMI, which includes snatching virtual red packets and special prices in the live streaming room, greatly enriches consumer experience. Specifically, IMI arouses consumers' social perception in two ways. On the one hand, consumers can obtain a substantial price or quantity discounts through IMI (e.g., coupons, prizes, and red packets), which cannot be obtained outside the live streaming room; the difference easily makes consumers perceive the existence of the community, thus stimulating consumer participation (Xue et al., 2020). On the other hand, adoption of interactive marketing tools (e.g., lucky wheel and limited-time lottery) can create a strong hedonistic atmosphere (Park and Lin, 2020). These immersive experiences help consumers form social presence. In fact, interactions in live-streaming platforms are more efficient than traditional forms; and as a result, the sense of social presence created is stronger. Hence, the following hypothesis is proposed:

**H1c: Interactions for obtaining monetary incentives (IMI) have a positive impact on social presence**.

### Consumer conformity

Consumer conformity can be defined as how consumers change their product evaluations, purchase intentions, and purchasing behaviors after receiving information regarding other people's product evaluations, purchase intentions, and purchase behaviors for the purpose of making their choices consistent with those of others (Lascu and Zinkhan, 1999). Based on Deutsch and Gerard (1955), this concept can be divided into two dimensions: normative and informational consumer conformity. The former acts in a consistent manner to conform with the positive expectations of another, i.e., purchasing certain products to be consistent with his or her own social group. The latter refers to the conforming to others purchase beliefs and decisions due to their knowledge and expertise.

In live-streaming e-commerce, normative consumer conformity is reflected in consumers' behaviors that conform to perceived expectations. For example, real-time interactions may enhance viewers' sense of belonging, make consumers more likely to internalize the opinion of the group and conform to the group's evaluation, and thus enhance social commerce engagement (Xue et al., 2020). As consumers can witness the active participation of other consumers through the bullet screen (i.e., “danmu” in Chinese, where viewers' comments and messages), consumers' purchase experiences are not isolated and become closer to shopping in offline settings. Moreover, it is difficult for consumers watching a live stream to resist the temptation of concerted large-scale group action, and consumer conformity in the live streaming room accelerates the process of consumer decision-making (Lu et al., 2016; Wongkitrungrueng and Assarut, 2018). Furthermore, the atmosphere and excitement created in live streaming rooms, the real-time interactions, and the continuous sense of participation brought about by the setting's constant engagement all combine to make live streaming shopping an addicting experience for consumers (Sun et al., 2019). These factors all work to make consumers feel a more direct and specific social connection, thereby encouraging them to behave in line with group expectations and guiding the emergence of consumer conformity.

The informational social influence of consumer conformity on the live streaming room is mainly reflected by the fact that consumers often purchase products based on the professional knowledge and product evaluation of others. In online shopping, the nature of the virtual marketplace prevents consumers from assessing the quality of a business or a product through face-to-face observation, and they cannot perceive the quality of a business or a product through personal experience (Smink et al., 2019). The information asymmetry between the two sides makes it difficult for consumers to fully understand the product, thus resulting in extreme uncertainty. In such cases, consumers are more likely to follow other people's purchase behaviors, which can lead to consumer conformity. When an individual's choice is consistent with that of the group, the perceived uncertainty and risk can be reduced. By browsing a shopping website, consumers can easily check the historical sales volume and the number of comments, which are often large enough to reflect useful information about previous buyers' behaviors. Consumers often make decisions based on such pieces of information (Lascu and Zinkhan, 1999).

In live streaming, there are two sources for consumers to obtain product information: streamers and other viewers. First, streamers not only provide product display and demonstration but also give consumers purchase guidance and hints to drive consumers to show conformity behavior consistent with others' expectations (Park and Feinberg, 2010). Second, non-business sources of product information, such as peer communication, have greater credibility and may suppress the mistrust of business sources (Boush et al., 1993). The virtual marketplace prevents consumers from perceiving the reliability of product value through personal experience, so consumers tend to conform to others' purchase beliefs and decisions because of their lack of knowledge and expertise. Based on the information presented above, the current research suggests that interactions in obtaining product information can promote consumer conformity. Hence, the following hypothesis is proposed:

**H2a: Interactions for obtaining product information (IPI) have a positive impact on consumer conformity**.

During a live stream, the interactions in the live streaming room enable consumers to observe the positive or negative evaluations of other consumers and their purchase behaviors and help consumers make consistent purchase decisions by reducing risks or increasing confidence. First, even in a live streaming situation, the lack of face-to-face interaction can lead customers to doubt the credibility of products, which increases the perceived risk of online shopping (Sun et al., 2019). When an individual's choice is consistent with that of the group, the perceived uncertainty and risk can be reduced. Second, consumers observe others' purchasing behaviors to determine whether the purchase decision is acceptable in society (Bearden et al., 1989). They usually consider being consistent with others as the safest and most economical way to buy and show purchase behavior consistency. Hence, the following hypothesis is proposed:

**H2b: Interactions for grasping the purchase dynamics of others (IPD) have a positive impact on consumer conformity**.

IMI may influence consumers' purchasing decisions and perceived risk by offering exclusive prices, coupons, and gifts. We propose that IMI affects consumer conformity in two ways. First, various coupons and seckilling in the live streaming room create a welcoming and lively atmosphere that makes it difficult for consumers to resist the temptation of large-scale group collaborative action, which enhances the tendency of conformity consumption (Huang et al., 2012). Second, perceived risk will decrease the customers' purchase intention, whereas deep discount could lower the customers' perceived risk to try a new product and thus increase the possibility of conformity behavior (Zhang and Yu, 2020). Hence, the following hypothesis is proposed:

**H2c: Interactions for obtaining monetary incentives (IMI) have a positive impact on consumer conformity**.

The social presence of live streaming may decrease consumers' doubts about products, thus promoting generation of conformity. First, the virtual community (e.g., live streaming room in the current research) satisfies the members' demand for emotional connections and belongingness as a social network (Park and Feinberg, 2010). In the specific environment of a live streaming room, the LSI for consumers to produce the warm feeling of others' presence prompts consumers to conform to the expectations of the group, and consumer conformity is expanded. Moreover, a sense of belonging in a group helps foster a more cohesive environment (Kim et al., 2004). In other words, the more a consumer feels to be a part of a group, the more likely they are to treat the group as trustworthy. Therefore, consumers tend to trust the expertise of reference group members and decrease their doubts about products, thus promoting generation of conformity. Hence, the following hypothesis is proposed:

**H3: Social presence has a positive impact on consumer conformity**.

### Purchase intention

Purchase intention determines whether consumers are likely to buy products in live-streaming e-commerce. It results from a strong agreement on the positive relationship between social presence and purchase intention, which can improve users' perceptions of online shopping security and their subsequent purchase attitude (Shin and Shin, 2011). Similarly, Sun et al. (2019) confirmed that social presence in an online shopping environment can make customers feel comfortable, and that such a positive feeling makes it easier for them to finalize purchase decisions. Moreover, it is easier to form a trustworthy relationship because social presence can shorten the perceived social distance between buyers and sellers (Lu et al., 2016). Furthermore, Wongkitrungrueng and Assarut (2018) divided consumer trust in the context of live streaming shopping into “trust in the seller” and “trust in the product”, and discovered that trust in the seller and product stemmed from customers' perceived social presence through live streaming, which led to their engagement behaviors. Consequently, their purchase intention will be stronger. Hence, the following hypothesis is proposed:

**H4a: Social presence has a positive impact on purchase intention**.

Conformity can lead to more efficient decision-making among consumers, which is why fostering conformity is a common marketing strategy used by advertisers to motivate consumers and encourage their product purchase intention (Lee and Park, 2008; Wu et al., 2017). In e-commerce, consumers often lack the knowledge and time to judge the attributes of a product and are therefore likely to imitate others instead. When other people's decisions are observable and continuous, consumer conformity often appears. In live-streaming e-commerce, other people's purchase behaviors are easily observed by potential buyers. Therefore, consumer conformity can greatly improve the possibility of consumer transactions in live-streaming e-commerce. Thus, the following hypothesis is proposed:

**H4b: Consumer conformity has a positive impact on purchase intention**.

### Experience products and search products

Based on knowledge of product quality, products can be classified into search products and experience products (Nelson, 1970). A search product is one that consumers can understand the quality and applicability of before they buy it. In contrast, the attributes of experience products cannot be obtained indirectly; instead, they must be evaluated through actual consumption or experience. Clothes, shoes, and accessories are examples of experience products, whereas mobile phones, laptops, and digital cameras are representatives of search products (Bei et al., 2004; Liu et al., 2016). In this study, the moderating role of product type is considered in our research model to examine whether the effects of consumers' social presence and conformity on their purchase intention is contingent on experience and search products.

Scholars usually consider the impact of product type in understanding whether a new marketing method has an impact on consumers' cognition and behavior. LSI eliminates time and space barriers and enables efficient consumer information acquisition, but an obvious weakness is that consumers cannot physically inspect, touch, and feel products at the time of purchase (Moon et al., 2008). Search and experience products in live streaming have significant differences in the process of information search and purchase decisions. During live streaming, consumers can obtain relevant information about the main attributes of the search product from the streamers without direct experience, relying less on the subjective evaluation and purchase decisions of the search product by other viewers. Besides, buyers of search products are greatly influenced by financial incentives, such as price concessions (Hsieh et al., 2005). Since the attributes of search products are easily identified online, consumers are most worried about whether the purchase price is favorable. This is different from the kind of worrying generated by evaluation of experience products. Overall, when consumers purchase search products, the impact of LSI on consumer purchasing decisions seems to be less strong compared than experience products.

On the other hand, because experience products are very challenging in terms of quality, price, and product specifications, consumers tend to rely on social influence before making a purchase decision (Hsieh et al., 2005). According to Bei et al. (2004), compared with search product buyers, experience product buyers place greater value on Internet-based information provided by other consumers and third parties. In particular, extreme reviews have a greater impact on experience products than on search products (Ghose and Ipeirotis, 2010); information cascades (i.e., a phenomenon where consumers select among multiple competing products) are more prominent for experience products than for search products (Liu et al., 2016). LSI provides an effective channel for experience product buyers to reduce perceived risks, that is, refer to the purchase behavior and evaluations of other purchasers, which then leads to consumer conformity. In addition, experience product buyers can also reduce the cost of perceived risks by participating in promotional activities in the live streaming room, thus promoting consumer consistency. Therefore, when consumers purchase experience products, the impact of LSI on consumer purchase decisions seems to be more obvious. Based on the above, the current research suggests that product type can moderate the influence of LSI on social presence and consumer conformity, which then affects purchase intention. Thus, the following hypotheses are proposed:

**H5a: When consumers purchase experience products (compared with search products), LSI have a greater impact on social presence and consumer conformity**.

**H5b: When consumers purchase experience products (compared with search products), social presence and consumer conformity have a greater impact on purchase intention**.

**Figure 1:**
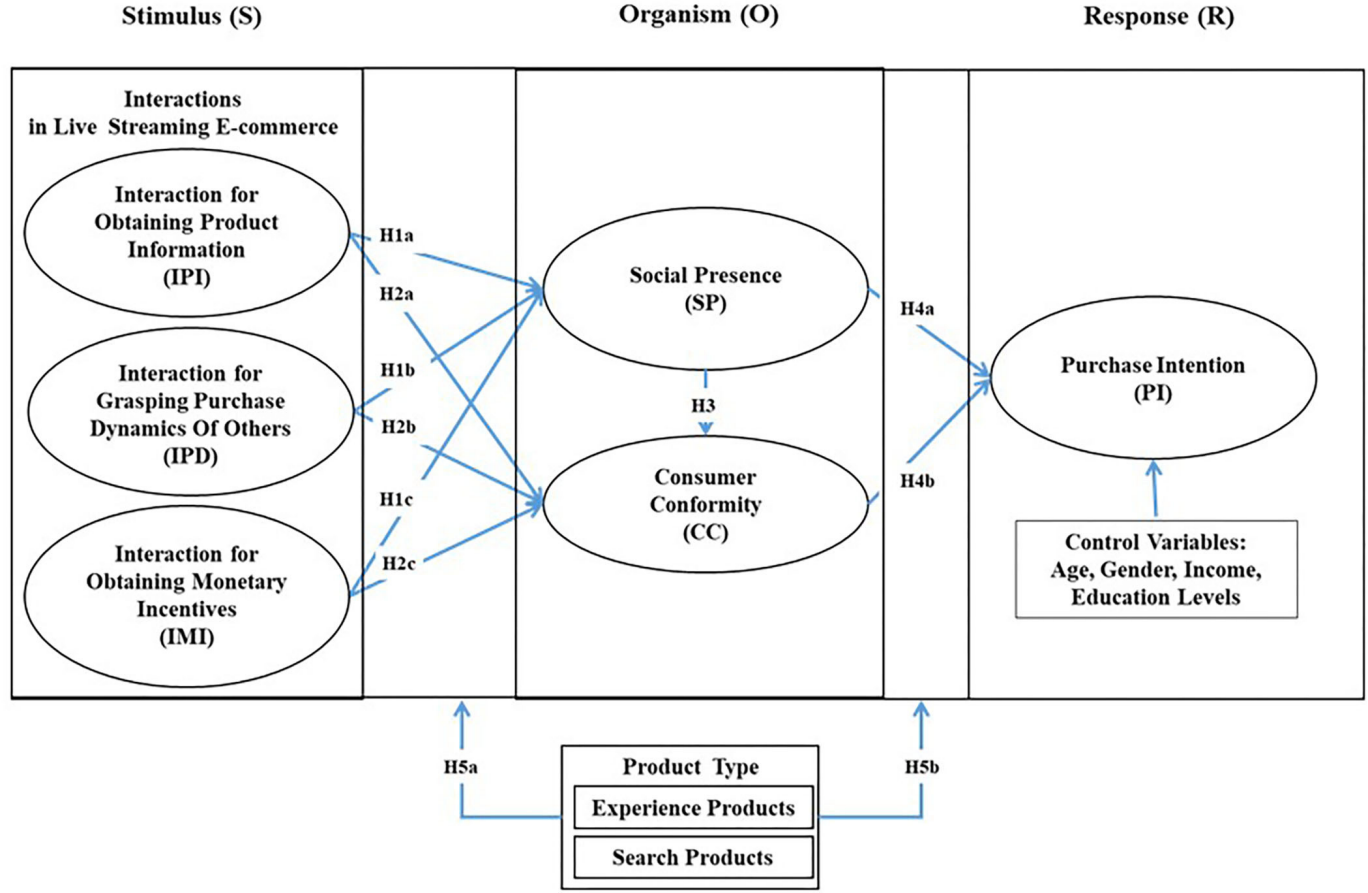
Research model.

On the basis of the S-O-R theory, we propose that LSI (including IPI, IPD, and IMI) can explain the formation of social presence and consumer conformity. Meanwhile, social presence can also influence consumer conformity, and both social presence and consumer conformity affect consumers' purchase intentions. Moreover, product type (i.e., search and experience products) moderates the relationships between them. In addition, we also employ age, gender, income, and education level as control variables. Finally, the research model is shown in Figure 1 below.

## Methodology

### Construct measurement

We designed a survey questionnaire and developed a survey instrument with items on a five-point Likert scale, ranging from complete disagreement to complete agreement. First, as mentioned in the introduction, LSI is operationalized as IPI, IPD, and IMI. Inspired by Jiang et al. (2019), Sun et al., 2019; Wongkitrungrueng and Assarut (2018), and Zhou et al. (2021), who build knowledge on the live streaming phenomenon, the measurement items of LSI are presented and adjusted to fit the context of live-streaming e-commerce. Second, social presence in live streaming is operationalized as the degree of interpersonal relationships felt by consumers, including human warmth and social communication, and its five-term measure is adapted from Gefen and Straub (2004), Ye et al. (2020), and Ou et al. (2014). Third, consumer conformity is operationalized through the effects of other consumers watching a live stream on consumers' purchase behavior and their desire to be consistent with others, and its five-term measure is adapted from Wu et al. (2017) and Lee and Park (2008). Fourth, purchase intention is operationalized as the intention of consumers to purchase products through live-streaming e-commerce, and its five-term measure is adapted from Chen et al. (2011); Sun et al. (2019), and Park and Lin (2020). The measurement items are shown in Table 1.

**Table 1:** Measurement items.

**Construct**	**Measurement items**	
Interaction for obtaining product information (IPI)	IPI1	When watching a live stream, the streamer's interesting explanation(s) makes it an addicting experience for them.
	IPI2	When watching a live stream, the streamer pays attention to adjusting the atmosphere during the product introduction.
	IPI3	When watching a live stream, I can leave messages to the streamer.
	IPI4	When watching a live stream, the streamer always answers questions immediately.
	IPI5	When watching a live stream, the streamer is always aware of my needs.
Interaction for grasping the purchase dynamics of others (IPD)	IPD1	When watching a live stream, I can see the number of viewers in the live streaming room.
	IPD2	When watching a live stream, I can see messages from other consumers.
	IPD3	When watching a live stream, I can see gifts from other consumers receive.
	IPD4	When watching a live stream, I can participate in discussions with other viewers.
	IPD5	When watching a live stream, I can see the purchases behaviors of other viewers.
Interaction for obtaining monetary incentives (IMI)	IMI1	When watching a live stream, I can enjoy exclusive low prices.
	IMI2	When watching a live stream, I have the opportunity to receive coupons.
	IMI3	When watching a live stream, I have the opportunity to draw prizes.
	IMI4	When watching a live stream, I have the opportunity to receive more gifts than other form.
	IMI5	When watching a live stream, I have the opportunity to extract red packets.
Social presence (SP)	SP1	When watching a live stream, there is a sense of human contact.
	SP2	When watching a live stream, I feel that I am not alone.
	SP3	When watching a live stream, I feel a sense of sociality.
	SP4	When watching a live stream, I feel a kind of humanized warmth.
	SP5	When watching a live stream, there is a sense of face-to-face communication.
Consumer conformity (CC)	CC1	When watching a live stream, I need to make a judgment by referring to the choices of other consumers.
	CC2	When watching a live stream, my choices are influenced by those of other consumers.
	CC3	When watching a live stream, other consumer's choices are always helpful for me.
	CC4	When watching a live stream, I think other consumers' choices are always right.
	CC5	When watching a live stream, I want to follow other consumers' choices.
Purchase intention (PI)	PI1	I will buy the products recommended by the live streaming platform.
	PI2	I will continue to buy products through live streaming e-commerce.
	PI3	I would like to recommend live streaming e-commerce to my family and friends.
	PI4	I have a good feeling about live streaming e-commerce.
	PI5	I experience greater enjoyment in shopping through live streaming e-commerce.

In addition, the moderating variable used in this study is product type. By investigating consumers' latest purchase experience, this study divided the respondents into two groups, namely, the experience product group and the search product group. This study referred to the division of experience products and search products by Bei et al. (2004), Cho (2015), Gupta et al. (2004), and Liu et al. (2016), and respondents purchasing experience products (e.g., clothing, shoes hats, foods, cosmetics, skincare products, toys, and games) were categorized into the experience product group. Respondents purchasing search products (e.g., household appliances and daily necessities) were categorized into the search product group. In particular, respondents selecting others were classified into one of the above groups by judging the types of specific product they had purchased. This is used to analyze how the relationships among interactions, social presence, consumer conformity, and purchase intention in live-streaming e-commerce change when purchasing different products.

### Data collection

The questionnaire was administered to residents in Shanghai, China. Shanghai was selected for the following two reasons. First, it is one of the top ten technologically matured cities in China, which ensures a stable and high-speed network environment for a fluent participation in LSI (PwC, 2020). Second, according to the report of Taobao Live Streaming Annual Report 2021, Shanghai has the largest number of live streaming e-commerce users in China (Liu and Li, 2021). Hence, we suggest that consumers in Shanghai can truly reflect the charm and efficiency of live-streaming e-commerce as a marketing tool. We visited department stores and coffee shops to find target respondents who have a recent shopping experience through live streaming e-commerce. The respondents were informed that the purpose of the survey was to explore whether LSI promotes both social presence and consumer conformity and thereby enhances consumers' purchase intentions.

Furthermore, participants who completed the questionnaire are from different industries and different age groups, but they all have a recent experience in shopping through live streaming e-commerce, which meets our survey requirements. Then, this questionnaire includes the respondents' demographic characteristics and their experience with live streaming e-commerce, followed by scales of the targeted variables in this study. All of the respondents were asked to recall their latest live streaming shopping experiences, the platforms they used, and the products they purchased. Based on the type of products they purchased during their latest live-streaming shopping experience, all the respondents were divided into two groups, namely, the experience product group and the search product group. After completing the questionnaire, the respondents received Starbucks coupons as rewards for their participation in this survey. Since the data collection was conducted in Shanghai and the original items were devised in English, we adopted the standardized method of back-translation to set up the questionnaire for the Chinese context. First, we invited a professional translator to translate our English version of the questionnaire into Chinese. Thereafter, another translator was assigned to back-translate the Chinese questionnaire into English to enhance translation equivalence. We carefully compared and proofread the two versions of the questionnaire to ensure no discrepancies between the two versions of the questionnaire. Finally, we used the Chinese version of the questionnaire to collect data in Shanghai. A total of 610 questionnaires were distributed, and 592 questionnaires were collected for a recovery rate of 96%. By scrutinizing all responses, omitting the same answer for all measurement questions, and excluding incomplete questionnaires, 576 valid responses were collected, which satisfied the minimum requirements.

### Descriptive analysis

In our sample, 92% of the respondents were under 40 years old, 46.4% of them were male and 53.6% were female. Among them, 74.7% had a monthly income between 5,000 and 15,000 RMB. The live streaming platforms they often used were Taobao, Douyin, Tmall, Kuaishou, Xiaohongshu, and Jingdong. In terms of sales volume, the top six categories of products they purchased from live streaming e-commerce platforms were clothing, shoes, hats, daily necessities, foods, cosmetics and skincare products, home appliances, and toys and games. The details are presented in Table 2 below.

**Table 2:** Survey respondent demographics.

**Measure**	**Items**	**Frequency**	**Percentage**
Age	Younger than 20 years old	28	4.9%
	20–30 years old	268	46.5%
	30–40 years old	234	40.6%
	Older than 40 years old	46	8.0%
Gender	Male	267	46.4%
	Female	309	53.6%
Income (Per month)	Less than 2000RMB	41	7.1%
	2000–5000RMB	49	8.5%
	5000–10000RMB	210	36.5%
	10000–15000RMB	220	38.2%
	More than 15000RMB per month	56	9.7%
Education level	Secondary school or below	27	4.7%
	Junior college (In process or graduated)	189	32.8%
	Bachelor (In process or graduated)	288	50.0%
	Master's degree or above	72	12.5%
Live streaming platform frequently used	https://taobaolive.taobao.com/	375	65.1%
	https://www.tmall.com/	163	28.3%
	https://jdlive.jd.com/	94	16.3%
	https://live.pinduoduo.com/	35	6.1%
	https://www.mogu.com/	29	5.0%
	https://www.xiaohongshu.com/	114	19.8%
	https://www.vip.com/	46	8.0%
	https://www.douyin.com/	320	55.6%
	https://live.kuaishou.com/	161	28.0%
	https://www.huya.com/	30	5.2%
	https://www.douyu.com/	63	10.9%
	https://www.huajiao.com/	25	4.3%
Products purchased through live streaming e-commerce	Clothing, shoes, and hats	346	60.1%
	Daily necessities	256	44.4%
	Food	298	51.7%
	Cosmetics and skincare products	289	50.2%
	Home appliances	147	25.5%
	Toys and games	140	24.3%
	Books and stationery	86	14.9%
	Jewelry and accessories	15	2.6%
	Others	7	1.2%

### Non-response bias

Non-response bias has always been an important issue that needs to be paid attention to in questionnaire survey research. In this study, we examine the problem of non-response bias that may exist during data collection. According to the test procedure proposed by Armstrong and Overton (1977), we judged whether early and late respondents had similar characteristics. First, we divided the 576 respondents evenly by time stamp into early and late respondent groups. Second, the Chi-Square test was performed to compare whether there were significant differences between the groups in demographic characteristics. As shown in Table 3, the results reveal that there is no significant difference between the groups in terms of age, gender, income, and educational level. Therefore, we suggest that non-response bias was not a serious problem for our data.

**Table 3:** Means and chi-square test results for nonresponse bias.

**Measure**	**Items**	**Number of early**	**Number of late**	**Chi-Squared**	***p*-value**
		**respondents**	**respondents**		
Age	Younger than 20 years old	15	13	1.277	0.735
	20–30 years old	126	142		
	30–40 years old	120	114		
	Older than 40 years old	24	22		
Gender	Male	138	129	0.969	0.325
	Female	147	162		
Income (Per month)	Less than 2,000 RMB	25	16	2.878	0.578
	2,000–5,000 RMB	22	27		
	5,000–10,000 RMB	105	105		
	10,000–15,000RMB	105	115		
	More than 15,000 RMB per month	28	28	2.587	0.460
Education level	Secondary school or below	12	15		
	Junior college (In process or graduated)	102	87		
	Bachelor (In process or graduated)	135	153		

## Results

Covariance-based structural equation modeling (CB-SEM) was conducted to evaluate our structural model. Using this method, correlations between variables can be appropriately determined and moderation effects can be examined. Following factor analysis, the normality test, common method bias test, and the structural model test were performed with SPSS 24 and AMOS 23.

### Reliability and validity of the measurement model

Internal consistency was evaluated using Cronbach's alpha and composite reliability (CR). Based on the guideline of Nunnally (1978), Cronbach's α value should be greater than 0.7 to verify the reliability, and all the CR values in Table 4 exceed 0.8, indicating good internal consistency. According to Fornell and Larcker (1981), the factor loading of each item should be greater than the threshold value of 0.6 and AVE values should be greater than the threshold value of 0.6. As shown in Table 4, all the indicators meet or exceed the above criteria. Table 5 indicates the descriptive statistics and correlations of the constructs. The square root of the AVE for each latent construct was greater than any correlation. Thus, all pairs of constructs are fully discriminant and support discriminant validity.

**Table 4:** Reliability and validity of the constructs.

**Constructs**	**Factor loading**	**CR**	**Cronbach's alpha**	**AVE**
Interaction for obtaining product information (IPI)	0.710	0.868	0.898	0.569
	0.786			
	0.751			
	0.721			
	0.800			
Interaction for grasping the purchase dynamics of others (IPD)	0.767	0.887	0.880	0.612
	0.859			
	0.704			
	0.785			
	0.788			
Interaction for obtaining monetary incentives (IMI)	0.764	0.865	0.869	0.562
	0.771			
	0.756			
	0.744			
	0.713			
Social presence (SP)	0.691	0.876	0.874	0.587
	0.730			
	0.821			
	0.826			
	0.755			
Consumer conformity (CC)	0.818	0.840	0.856	0.515
	0.751			
	0.586			
	0.743			
	0.670			
Purchase intention (PI)	0.798	0.870	0.881	0.573
	0.852			
	0.718			
	0.705			
	0.700			

**Table 5:** Descriptive statistics and correlations.

**Constructs**	**Mean**	**Std. Dev**.	**IPI**	**IPD**	**IMI**	**SP**	**CC**	**PI**
IPI	3.505	0.984	0.754					
IPD	3.611	0.981	0.323^**^	0.782				
IMI	3.464	0.960	0.340^**^	0.331^**^	0.750			
SP	3.574	0.902	0.523^**^	0.509^**^	0.516^**^	0.766		
CC	3.649	0.913	0.561^**^	0.563^**^	0.372^**^	0.612^**^	0.718	
PI	3.439	0.877	0.451^**^	0.452^**^	0.372^**^	0.591^**^	0.611^**^	0.757

Significance levels:

^***^p < 0.001;

** p < 0.01;

^*^p < 0.05.

IPI, interaction for obtaining product information; IPD, interaction for grasping purchase dynamics of others; IMI, interaction for obtaining monetary incentives; SP, social presence; CC, consumer conformity; PI, purchase intention.

### Normalcy test

To check whether the data are normally distributed, two normalcy tests were conducted. First of all, the distribution of all variables, except for control variables, can be examined by the Skewness and Kurtosis test for normality. Correspondingly, the Skewness values were between −0.798 and −0.25 and Kurtosis values were between −1.077 and 0.273, and were both well within the acceptable threshold of ±2, suggesting the data are normally distributed (Kunnan, 1998). Moreover, the Shapiro-Wilk tests for each variable were performed, and the results did not reject the hypothesis of a normal distribution (D'Agostino and Balanger, 1990; Royston, 1991).

### Common method bias

Two statistical tests were conducted to assess the potential threat of common method bias. First, we conducted Harman's one-factor test using the guidelines of Podsakoff et al. (2003). The results of loading all observable items into one factor suggest a total variance of 35.2%, which is less than 40% and suggests that common method bias was overall not a serious issue in this research (Podsakoff et al., 2003; Cui et al., 2019). Furthermore, Bartlett's test of sphericity is 5,209.039 (*p* < 0.001), and the Keyser-Meyer-Olkin (KMO) measure of the sampling adequacy of the correlation matrix for factor analysis is 0.941 (exceeds the standard of 0.8), indicating the dataset is adequate (Kaiser, 1974). In addition, we also use the full-collinearity test of Kock (2015) to check for common method bias. The results of SmartPLS 3.0 show that the pathological variance inflation factor (VIF) ranged from 1.295 to 2.171, which indicates that common method bias is not a serious issue in our study.

### Structural model

Exploratory factor analysis (EFA), confirmatory factor analysis (CFA), and SEM analysis are conducted to evaluate the research model's goodness-of-fit indicators. A number of 288 respondents were drawn randomly from the total number of 576 to conduct the EFA and the remaining 288 cases were retained for the CFA. First, in order to confirm the reliability and validity of the measurement of LSI, this study conducts EFA and CFA. The results of the EFA show that the identified appropriateness of LSI measurement has been verified by KMO and Bartlett's test, its 15 items loaded clearly across the three LSI factors, and the total variance explained by all the constructs is above 60%, which confirms discriminant validity (Chin, 1998; Hair et al., 2006). Moreover, the results of the CFA also indicate that the model fit for LSI measurement is within the cut-off range (χ^2^= 116.351, df = 87, *p* < 0.001, GFI = 0.951, AGFI = 0.933, NFI = 0.947, IFI = 0.986, TLI = 0.983, CFI = 0.986, and RMSEA = 0.034), and standardized factor loadings vary between 0.71 and 0.86, suggesting good convergence of the construct.

**Figure 2:**
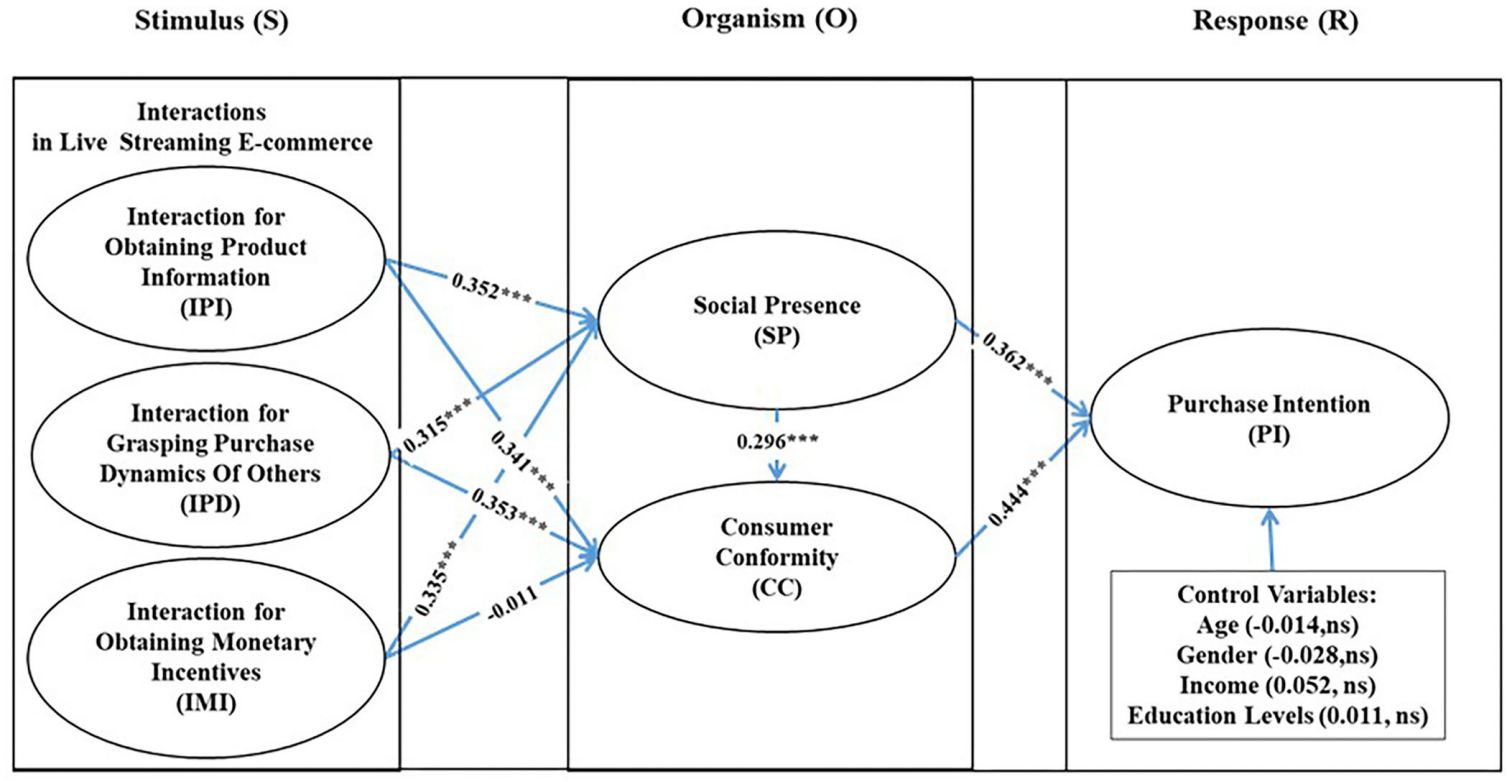
Results of the structural model.

Based on a full sample, Figure 2 depicts the full-path diagram and Tables 6, 7 show the results of overall model fit and SEM, respectively. Based on the guideline of Hair et al. (2014), a coefficient of determination value (*R*^2^) above 0.2 is considered relatively high and acceptable (Hair et al., 2014). As shown in Figure 2, the *R*^2^ values for social presence, consumer conformity, and purchase intention are 0.586, 0.644, and 0.555, respectively, which indicate that the SEM results are acceptable. Moreover, Table 6 indicates that the overall fit of the model is in the acceptable range (χ^2^ = 951.761, df = 548, *p* < 0.001, GFI = 0.913, AGFI = 0.9, NFI = 0.907, IFI = 0.958, TLI = 0.955, CFI = 0.958, and RMSEA = 0.056). Accordingly, the above suggests that the demonstrated SEM model fits the data well.

**Table 6:** Test of model fit.

		**df**	**Δx^2^/df**	**SRMR**	**GFI**	**AGFI**	**NFI**	**IFI**	**TLI**	**CFI**	**RMSEA**
Suggested			1~3	<0.05	>0.9	>0.9	>0.9	>0.9	>0.9	>0.9	<0.08
Actual	951.761	548	1.737	0.024	0.913	0.900	0.907	0.958	0.955	0.958	0.056

**Table 7:** Results of the structural model and hypothesis testing.

**Hypothesis**	**Causal Path**			**Estimate**	**S.E**.	**C.R**.	***p***	**Path coefficient**	**R^2^**	**Results**
H1a	IPI	→	SP	0.303	0.038	7.986	^***^	0.352	0.586	Supported
H1b	IPD	→	SP	0.242	0.033	7.339	^***^	0.315		Supported
H1c	IMI	→	SP	0.246	0.032	7.584	^***^	0.335		Supported
H2a	IPI	→	CC	0.375	0.051	7.362	^***^	0.341	0.644	Supported
H2b	IPD	→	CC	0.346	0.044	7.802	^***^	0.353		Supported
H2c	IMI	→	CC	−0.010	0.041	−0.245	0.806	−0.011		Rejected
H3	SP	→	CC	0.378	0.077	4.925	^***^	0.296		Supported
H4a	SP	→	PI	0.452	0.073	6.174	^***^	0.362	0.555	Supported
H4b	CC	→	PI	0.444	0.057	7.697	^***^	0.444		Supported

Significance levels:

*** p < 0.001;

^#x0002A;*^p < 0.01;

^*^p < 0.05.

IPI, interaction for obtaining product information; IPD, interaction for grasping purchase dynamics of others; IMI, interaction for obtaining monetary incentives; SP, social presence; CC, consumer conformity; PI, purchase intention.

The SEM results show that all the path coefficients are statistically significant, except for that of H2c (shown in Table 7). IPI (path coefficient = 0.352, *p* < 0.001), IPD (path coefficient = 0.315, *p* < 0.001), and IMI (path coefficient = 0.335, *p* < 0.001); all significantly affect social presence (SP), thus, H1a, H1b, and H1c are strongly supported. Moreover, IPI (path coefficient = 0.341, *p* < 0.001) and IPD (path coefficient = 0.353, *p* < 0.001) both have a significant positive effect on consumer conformity (CC), thus supporting H2a and H2b, respectively. Furthermore, social presence (SP) has a significant positive effect on consumer conformity (CC, path coefficient = 0.295, *p* < 0.001), thus supporting H3. Finally, purchase intention (PI) is significantly positively influenced by social presence (SP, path coefficient = 0.359, *p* < 0.001) and consumer conformity (CC, path coefficient = 0.444, *p* < 0.001), thereby supporting H4a and H4b, respectively.

### The moderating effect of product type

To examine the moderating effect of product type, a multigroup SEM analysis was performed. The purpose of the analysis is to evaluate the fitness of a preset model among different groups, i.e., to evaluate whether the structural models are equivalent among different groups or have parameter invariance (Byrne, 2010). The principle of multigroup SEM analysis is to divide the original single covariant structure into several parallel covariant structures, after which the equivalence of the covariant structures is evaluated. In the current research, product type is used as the moderating variable for the multigroup analysis, for which an unconstrained model (without any model parameter limitation) and a constrained model (with limited structure coefficient equals) should be established.

Respondents purchasing experience products such as clothing, shoes, hats, foods, cosmetics and skincare products, toys and games, and jewelry and accessories were classified under the experience product group. Respondents purchasing search products such as daily necessities, home appliances, and books and stationeries were classified under the search product group. Of the 576 respondents, 360 purchased experience products (62.5%) and 216 bought search products (37.5%) in their latest live stream shopping experience. We tried to determine whether there was a significant difference in the path between the experience product and search product groups. In comparing the constrained model with the unconstrained model, if the *x*^2^ difference test reaches a significant level, it indicates that the models are not equal and that there are significant differences between the two product groups. As shown in Table 8, the fitting indexes of the constrained model and unconstrained model reach the ideal value, indicating that the two models are acceptable. The *x*^2^ difference between the unconstrained and constrained models is significant (Δ*x*^2^ = 102.489, *p* < 0.001), indicating a significant difference between the two groups in relation to the moderating role of product type.

**Table 8:** Differences between the unconstrained and constrained models.

		**df**	**Δx^2^/df**	**Δx^2^**	**SRMR**	**RMSEA**	**GFI**	**NFI**	**IFI**	**TLI**	**CFI**
Suggested			<3		<0.08	<0.08	>0.90	>0.90	>0.90	>0.90	>0.90
Unconstrained Model	1009	786	1.284	102.489	0.033	0.022	0.902	0.906	0.978	0.975	0.978
Constrained Model	1112	795	1.398		0.036	0.026	0.893	0.897	0.968	0.965	0.968

As shown in Table 9, multigroup SEM was performed to compare the hypothesized relationships between the experience product and search product groups. The influence of IPI on social presence (SP) is different between the two groups (t = −2.325, *p* < 0.05); the path coefficient is 0.492 (*p* < 0.001) in the experience product group and 0.158 (*p* < 0.05) in the search product group, indicating that the influence is more significant in the former than in the latter. The influence of IPD on social presence (SP) is different between the two groups (t = −2.122, *p* < 0.05); the path coefficient is 0.484 (*p* < 0.001) in the experience product group and 0.146 (*p* < 0.05) in the search product group, indicating that the influence is more significant in the former than in the latter. The influence of IPI on consumer conformity (CC) is different between the two groups (t = −2.099, *p* < 0.05). Here, the path coefficient is 0.461 (*p* < 0.001) in the experience product group and 0.228 (*p* < 0.01) in the search product group, indicating that the influence is more significant in the former than in the latter. The influence of IPD on consumer conformity (CC) is different between the two groups (t = −2.441, *p* < 0.05); the path coefficient is 0.478 (*p* < 0.001) in the experience product group and 0.179 (*p* < 0.05) in the search product group, revealing that the influence is more significant in the former than in the latter. However, the influence of IMI on social presence (SP) is different between the two groups (t = 5.129, *p* < 0.001). Here, the path coefficient is 0.144 (*p* < 0.01) in the experience product group and 0.553 (*p* < 0.001) in the search product group, indicating that the influence is more significant in the latter than in the former. Meanwhile, the effect of IMI on consumer conformity (CC) is significant in neither group. On the basis of the discussion presented above, H5a is partially established.

**Table 9:** Results of the multigroup structural equation modeling.

**Hypothesis**	**Causal path**			**Experience product group**			**Search product group**		**Critical ratio**		**Results**
				**Path coefficient**	***p***		**Path coefficient**	***p***	**T**	***p***	
H5a	IPI	→	SP	0.492	^***^	>	0.158	^*^	−2.325	^*^	Supported
	IPD	→	SP	0.484	^***^	>	0.146	^*^	−2.122	^*^	Supported
	IMI	→	SP	0.144	^**^	<	0.553	^***^	5.129	^***^	Rejected
	IPI	→	CC	0.461	^***^	>	0.228	^**^	−2.099	^*^	Supported
	IPD	→	CC	0.478	^***^	>	0.179	^*^	−2.441	^*^	Supported
	IMI	→	CC	−0.005	0.891	/	−0.024	0.822	−0.178	0.859	Rejected
H5b	SP	→	PI	0.523	^***^	>	0.201	^*^	−2.616	^**^	Supported
	CC	→	PI	0.482	^***^	>	0.187	^*^	−1.983	^*^	Supported

Significance levels:

*** p < 0.001;

** p < 0.01;

* p < 0.05.

IPI, interaction for obtaining product information; IPD, interaction for grasping purchase dynamics of others; IMI, interaction for obtaining monetary incentives; SP, social presence; CC, consumer conformity; PI, purchase intention.

Table 9 indicates that H5b is established. The influence of social presence (SP) on purchase intention (PI) is different between the two groups (t = −2.616, *p* < 0.01); the path coefficient is 0.523 (*p* < 0.001) in the experience product group and 0.201 (*p* < 0.05) in the search product group, indicating that the influence of the former is more significant than that of the latter. The influence of consumer conformity (CC) on purchase intention (PI) is different between the two groups (t = −1.983, *p* < 0.05). Here, the path coefficient is 0.482 (*p* < 0.001) in the experience product group and 0.187 (*p* < 0.05) in the search product group, indicating that the influence of the former is stronger than that of the latter. On the basis of the discussion presented above, H5b is supported.

## Discussion

### Findings

The interactions that occur in live streaming e-commerce platforms overcome the disadvantages of traditional e-commerce and provide consumers with an interesting shopping experience. This research analyzes the relationships between LSI, social presence, consumer conformity, and purchase intention. The moderating role of product type in these relationships is also explored.

Several findings can be derived from the empirical results. First, the validity of the three-purpose classification of LSI was revealed. Interactions are regarded as the major characteristic of live-streaming e-commerce and explain live-streaming e-commerce's success. IPI, IPD, and IMI are the three classes of interactions examined in this research. The results show that IPI and IPD can exert a direct impact on social presence and consumer conformity in the live streaming e-commerce context, which is consistent with previous research (Jiang et al., 2019). This finding can be attributed to interactions being able to not only answer consumers' doubts about products but also increase the familiarity, intimacy, and warmth among them. The creation of positive emotions gives consumers the sense that they are part of a group and truly interacting with others, thus enabling offline shopping to evoke a pleasant experience (Tajvidi et al., 2017; Wang et al., 2019). Through LSI, consumers are no longer isolated, and people's behaviors tend to influence others who, in striving to stay in step with the herd, create conformity. The findings reveal that IMI has a positive impact on social presence but that it has no impact on consumer conformity. In line with Zhang et al. (2014), this implies that the formation of consumer conformity in live streaming comes mainly from the information and emotional values engendered by interactions and not from monetary incentives.

Second, our model proves an effective integration of social presence, consumer conformity, and purchase intention. The findings suggest that social presence positively influences consumer conformity, and that, in turn, social presence and consumer conformity enhance purchase intention, which was confirmed in a previous study (Li, 2019). This is because in live streaming, although consumers cannot physically handle the products, frequent interactions and communication can create an almost face-to-face feeling, which can dispel consumers' doubts and convince them to make purchase decisions.

Third, the multigroup analysis indicates that product type can moderate the above relationships because of the different attributes of experience and search products. Concerning the experience product group, IPI and IPD have a more significant impact on social presence, consumer conformity, and purchase intention. In comparison, for the search product group, the IMI in the search product group has a more significant effect on social presence. This may be caused by potential factors, such as product price. IMI is often accompanied by price concessions. For search products, buyers are greatly influenced by financial incentives, and lower product prices stimulate consumers' sense of social presence (Hsieh et al., 2005).

In view of these differences, live streaming marketing can be seen as more suitable for selling experience products because it simultaneously merges the opinions of streamers and consumers, which undoubtedly reduces the difficulty of assessing an experience product purchase. Thus, consumers who purchase experience products also expect more from live streaming. When it comes to search products, the channel from which to obtain information is relatively simple; therefore, incentive policies and measures to promote the sales of these products are particularly important in live streaming. This is a new perspective for marketers who need to consider what products are more suitable for live-streaming marketing and find more efficient ways to promote live-streaming e-commerce. These findings enable us to observe and predict more accurately the consumer decision-making process in live-streaming e-commerce in place to achieve a fruitful marketing outcome.

### Implications

As one of the latest marketing modes, live streaming e-commerce's business value surpasses that of traditional e-commerce by greatly improving shopping experience. This study created a model through which we can utilize interactions in live streaming e-commerce platforms to increase social presence and consumer conformity, which in turn, enhances consumers' purchase intention. By investigating the relationships among LSI, social presence, consumer conformity, and purchase intention, the current study aims to fill a gap and contribute to the e-commerce literature in four ways.

The first critical contribution of this study is that it extended and enriched recent efforts in the literature to understand interactions in live streaming e-commerce. The existing literature on live streaming interaction has often focused on person interactivity and machine interactivity. However, with the integration of live streaming e-commerce, a richer consumer experience has been created, and LSI can no longer be simply divided by participants. Compared to past studies on interactions in this context, which are mostly classified according to the object of interactions (Sun et al., 2019), this study developed a new measurement for LSI based on a CMC interaction model and consumers' motivation to participate in live streaming, and proposed three dimensions: IPI, IPD, and IMI. In particular, we employed IMI as a dimension of monetary incentive interactions and verified its role in increasing social presence. This study is consistent with Garnefeld et al. (2012) who proposed that monetary incentives can enhance community member's intention to participate. In view of the effectiveness of this interaction framework, we recommend that live streaming platforms should concentrate on increasing interactivity of live streaming e-commerce. By developing interactive tools, consumers could enjoy more shopping experiences and emotional value.

The second contribution is that this study advances the understanding of consumers' purchase intention in the context of live streaming. In previous studies, scholars have attempted to reveal factors influencing consumers' purchase intention from different aspects, such as online streamers (Jiang et al., 2020), virtual gifts (Guan et al., 2022), and product-content fit (Park and Lin, 2020). Moreover, technology features (i.e., interactions, visibility affordance, meta voicing affordance, and guidance shopping affordance) have been considered as a factor in consumers' purchase intentions through live-streaming e-commerce (Sun et al., 2019). However, the studies did not focus on the effects of live streaming interactions on consumers' purchase intention. This study demonstrates that LSI in e-commerce platforms can positively influence consumers' purchase intention through social presence and consumer conformity. Therefore, marketers who are yet to adopt live streaming marketing should consider doing so in order to improve their sales performances. Meanwhile, marketers who are already doing live broadcast marketing should consider how they can improve LSI. For example, by comprehensive mobilization of audiovisual tools, the interactive effect on live streaming can be heightened to improve the speed of providing responses to consumers' questions and to improve their live shopping experiences. Moreover, streamers must have a complete understanding of the products they are promoting to facilitate better product presentation and eliminate consumers' uncertainties.

The third contribution of this study is that we apply and integrate the two theories of social presence and consumer conformity with an examination of consumers' purchase intention in the live streaming e-commerce context. First, the social presence theory has been widely applied in online consumption research (Botha and Reyneke, 2016; Fang et al., 2018; Jiang et al., 2019), but only a few studies have been conducted on live streaming interactions. Second, although the theory of consumer conformity has been previously applied in e-commerce research, it has not been applied in live streaming e-commerce research. Therefore, this study expands the application of the previously mentioned two theories in live-streaming e-commerce. It also confirms the positive influence of social presence on consumer conformity and explains the potential incentives for consumers' purchases in live-streaming e-commerce. These results indicate that the goals of improving social presence and enhancing consumer conformity must be considered in live-streaming e-commerce development strategies.

The fourth contribution of this study is that it pays more attention to product factors, which have been ignored in previous studies. Many past studies have only focused on consumer behaviors in live streaming e-commerce from the consumer's perspective; however, in real-world business environments, consumer behaviors are inevitably affected by product factors. Therefore, this study introduces product type as a moderating variable and confirms the differences in the path coefficients of different product types by empirical research. Consumers who seek experience products require more interactions with streamers and other consumers to obtain sufficient information; thus, social presence and consumer conformity are more significant in purchasing these products. In comparison, consumers looking for search products tend to put more emphasis on price, which can more significantly affect social presence. Marketers should thus consider a product's attributes when determining their live streaming marketing strategies and create different live-streaming marketing plans depending on product type. For experience products, first, streamers probably require extra efforts to help consumers conduct a detailed and comprehensive review of experience products, and provide more attribute-based and experience-based product information in live streaming to improve consumers' perceived usefulness. Second, the key attributes of experience products are subjective. Streamers should encourage consumers to post real personal experience through real-time interaction on the live streaming platform, so as to reduce consumers' uncertainty. Third, by increasing interaction methods to create a good interactive atmosphere, consumers can improve their sense of social presence and promote their purchase decision. On the other hand, providing more attribute-based product information, increasing preferential product pricing, and offering group purchase pricing are more effective strategies in guiding the purchase of a search product.

### Limitations and future research directions

The limitations of this study and future directions for research can be addressed as follows. First, this study suggests that while LSI increases social presence and consumer conformity, other factors should also be considered in future studies, such as consumers' gender, shopping frequency, and shopping motivations. Second, the level of development of e-commerce and cultural background may vary from region to region, and this study was conducted in Shanghai, China. Thus, the results of this study may not be generalizable to regions that differ greatly from Shanghai. In future studies, regional factors should be taken into consideration for collection of more accurate data for analysis and comparison.

## Data availability statement

The raw data supporting the conclusions of this article will be made available by the authors, without undue reservation.

## Ethics statement

Ethical review and approval was not required for the study on human participants in accordance with the local legislation and institutional requirements. Written informed consent for participation was not required for this study in accordance with the national legislation and the institutional requirements.

## Author contributions

FL: conceptualization, methodology, formal analysis, writing—original draft, writing—review and editing, and supervision. HZ: conceptualization, methodology, formal analysis, data curation, writing—original draft, and writing—review and editing. XD: investigation and writing—review and editing. YW: investigation, formal analysis, and writing—review and editing. All authors contributed to the article and approved the submitted version.

## Funding

This work was supported by the Humanities and Social Sciences Foundation of the Ministry of Education of China (Grant No. 21YJC630076).

## Conflict of interest

The authors declare that the research was conducted in the absence of any commercial or financial relationships that could be construed as a potential conflict of interest.

## Publisher's note

All claims expressed in this article are solely those of the authors and do not necessarily represent those of their affiliated organizations, or those of the publisher, the editors and the reviewers. Any product that may be evaluated in this article, or claim that may be made by its manufacturer, is not guaranteed or endorsed by the publisher.
